# A metamaterial unit-cell based patch radiator for brain-machine interface technology

**DOI:** 10.1016/j.heliyon.2024.e27775

**Published:** 2024-03-11

**Authors:** Emtiaz Ahmed Mainul, Md Faruque Hossain

**Affiliations:** Department of Electronics and Communication Engineering, Khulna University of Engineering & Technology, Khulna-9203, Bangladesh

**Keywords:** Implantable antenna, ISM band, Biotelemetry, Inset-fed, Tissue phantom, In-vivo, Brain-machine interface, Specific absorption rate, Metamaterial

## Abstract

This paper presents a novel approach to the design of a brain implantable antenna tailored for brain-machine interface (BMI) technology. The design is based on a U-shaped unit-cell metamaterial (MTM), introducing innovative features to enhance performance and address specific challenges associated with BMI applications. The motivation behind the use of the unit-cell structure is to elongate the electric path within the antenna patch, diverging from a reliance on the electrical properties of the MTM. Consequently, the unit cell is connected to an inset-fed transmission line and shorted to the ground. This configuration serves the dual purpose of reducing the size of the antenna and enabling resonance at the 2.442 GHz band within a seven-layer brain phantom. The antenna is designed using a FR-4 substrate ($\varepsilon_{r}$ = 4.3 and tan δ = 0.025) of 1.5 mm thickness, and it is coated with a biocompatible polyamide material ($\varepsilon_{r}$ = 4.3 and tan δ = 0.004) of 0.05 mm thickness. The proposed antenna achieves a compact dimension of 20 × 20 × 1.6 ${mm}^{3}$ (0.338 × 0.338 × 0.027 $\lambda_{g}^{3}$) and demonstrates a high bandwidth of 974 MHz with its gain of −14.6 dBi in the 2.442 GHz band. It also exhibits a matched impedance of 49.41-j1.32 Ω in the implantable condition, corresponding to a 50 Ω source impedance. In comparison to a selection of relevant research works, the proposed antenna has a low specific absorption rate (SAR) of 218 W/kg and 68 W/kg at 1g and 10g brain tissue standards, respectively. An antenna prototype has been fabricated and measured for return loss in both free space and in-vivo conditions using sheep's brain. The measurement results are found to be in close agreement with the simulation results for both conditions, showing the practical applicability of the proposed antenna for BMI applications.

## 1 Introduction

More recently, advancements in neuroengineering have paved the way for brain-machine interfaces (BMIs), enabling a direct connection between the brain and external devices. This progress has opened up new avenues for applications such as neuroprosthetics, cognitive enhancement, and rehabilitation. BMIs can enhance the immersive experience in virtual and augmented reality applications by directly translating neural signals into actions within the virtual environment, creating more natural and engaging user interactions. Playing a pivotal role in facilitating this brain-device communication are implanted antennas. By merging the biological and technological domains, these antennas enable the transfer of neural impulses from the brain to an external receiver, as well as the exchange of data or control signals from the external device to the brain.

In designing antennas for BMI applications, several specific criteria must be taken into account, including antenna miniaturization, high bandwidth, substantial gain, low specific absorption rate (SAR), optimal link budget, and most importantly, the antenna needs to be operated in a dedicated frequency band. Commonly employed frequency bands for implantable antennas are the Medical Implant Communications Services (MICS) band (402–406 MHz) and the Industrial, Scientific, and Medical (ISM) bands, encompassing ranges such as 433–434 MHz, 868–868 MHz, 902–928 MHz, and 2.4–2.4835 MHz [[1], [2], [3], [4]]. Among these, the high-frequency ISM band (2.4 GHz) is preferred for a high-speed wireless link between the internal neural recording unit and the external unit for processing and decoding the neural activities [ [5]]. Besides, some countries have approval for ultra-wideband frequency bands (UWB, 3.1–10.6 GHz) to facilitate high-quality transmission [ [2]]. Additionally, these antennas need to be powered wirelessly, necessitating a directional radiation pattern for the antenna [ [5]].

Numerous endeavours have been made to design implantable antennas for brain applications [[5], [6], [7], [8], [9]]. For instance, a compact, circular-shaped brain implantable antenna with dimensions of 39.3 ${mm}^{3}$ exhibited a bandwidth of 14.9% and a gain of −20.75 dBi at the 2.4 GHz frequency [ [5]]. However, a circular-shaped antenna is considered better than a rectangular-shaped antenna in brain implants, even though the antenna exhibited a low radiation efficiency of 0.24%. Another study presented a defected ground structure (DGS) and rectangular-shaped antenna with dimensions of 6 × 9.55 × 0.524 ${mm}^{3}$, exhibiting high specific absorption rates (SAR) of 682 W/kg for 1g and 77.1 W/kg for 10g standards [ [6]]. Although DGS assists in achieving compactness, precision is crucial, and any manufacturing variations can affect the performance of the DGS-based antenna. A different study featured a rectangular-shaped and coaxial-fed antenna with dimensions of 10 × 10 × 1.5 ${mm}^{3}$, operating at 2.475 GHz. This antenna demonstrated a broadside radiation pattern, albeit with limited bandwidth (80 MHz) and a gain of −25 dBi [ [7]]. Artificial tissue-emulating (ATE) materials were fabricated to measure the proposed antenna, although it was challenging to obtain the perfect permittivity and loss tangent of each semi-solid ATE. Meanwhile, a small footprint of 12 × 7 ${mm}^{2}$ Vivaldi antenna was presented with vertical slots, operating in the 4 GHz band [ [8]]. The antenna demonstrated a wide bandwidth of 2 GHz and a peak gain of −15.7 dBi. However, the resonance frequency of the antenna was 4 GHz, which is considered slightly high for an implantable device. When electromagnetic fields associated with high resonance frequencies interact with biological tissues, there is a risk of energy absorption, leading to an increase in tissue temperature [ [1]]. The consequences of elevated temperatures within the human body include tissue damage, physical pain, etc. A meandered planar-inverted-F antenna (PIFA) with a total volume of 11 × 20.5 × 1.8 ${mm}^{3}$ was proposed for triple-band operation (401–406 MHz, 902–928 MHz, and 2400–2483.5 MHz), exhibiting comparatively low gains of −43.6 dBi, −25.8 dBi, and −20.1 dBi, respectively [ [9]]. A homogeneous liquid phantom was used to measure the antenna, and the ISM band (2400 MHz) shifted to a slightly higher band in that test. Various other implantable antenna configurations with different characteristics have also been proposed [[10], [11], [12], [13]]; however, no single antenna design excels in all parameters. This has led to the pursuit of designing an implantable antenna that balances compact dimensions, high bandwidth, substantial gain, and low SAR.

Antenna miniaturization in the context of brain-machine interface applications is crucial for achieving unobtrusive integration, enhancing biocompatibility, and optimizing the performance of implantable devices within the confined and sensitive environment of the human brain. Therefore, the drive for antenna miniaturization primarily stems from the space constraints of implantable medical devices (IMDs), the desire for lower power consumption, and the need to minimize biological effects. Low-frequency signals (longer wavelengths) typically require larger antennas for effective radiation, while high-frequency signals (shorter wavelengths) require smaller antennas, unless specific design engineering techniques are applied to alter this relationship. Achieving antenna compactness often involves employing substrates with high permittivity, which reduces the guided wavelength and consequently lowers the resonance frequency. A substrate with a very high permittivity, zirconia material ($\varepsilon_{r}$ = 27, σ ≈ 0, tanδ ≈ 0), with a thickness of 0.05 mm, was used for the purpose of miniaturization in [ [12]]. In contrast, a substrate of higher permittivity will affect radiation efficiency by converting antenna input power into surface waves [ [13]]. Techniques like incorporating shorting pins between the radiating patch and ground [ [4]] or lengthening current paths within the antenna radiator have been employed to minimize antenna size while maintaining optimal performance [ [9,11]]. Furthermore, compact-sized implantable antennas often exhibit limited bandwidth. On the other hand, a large bandwidth is necessary to support a high data rate as well as to minimize the effect of frequency detuning caused by different body tissues and the orientation of the antenna in the same body tissue [ [10,14]].

In terms of biocompatibility, given the conductive nature of human tissue, precautions must be taken to avoid tissue damage due to short circuits between the antenna metal and tissue. Biocompatible superstrates like Teflon, Alumina, Zirconia, PEEK, and MACOR can be used to ensure tissue safety [ [4]]. A thin (0.05 mm) layer of polyamide ($\varepsilon_{r}$ = 4.3 and tan δ = 0.004) superstrate can be employed to address biocompatibility issues [ [3]]. Additionally, to comply with safety standards, it is also vital to keep the specific absorption rate (SAR) within allowable limits regulated by IEEE C95.1–1999 and IEEE C95.1–2005 [ [2,9]].

In the realm of antenna development, a growing trend involves the integration of metamaterial (MTM) structure onto the antenna's patch or ground. These structures exhibit negative effective permittivity and permeability values at specific frequencies, effectively enlarging the size of the radiating element beyond its physical dimensions and enhancing gain and directivity by manipulating electromagnetic wave propagation. Numerous works have explored this avenue [[15], [16], [17], [18]], incorporating unit cell metamaterial structures on the antenna patch or ground to improve gain and directivity. For example, a gain enhancement of approximately 2 dB at 915 MHz and 1.5 dB at the 2450 MHz frequency band were observed when incorporating the metamaterial (MTM) into the antenna structure [ [17]]. Another implantable antenna was designed with two split-ring resonators to achieve a positive gain of 4.816 dB at the 2.4–2.5 GHz frequency band [ [18]].

Therefore, the primary objective of this work is to design an electrically small antenna suited for brain-machine interface (BMI) technology. Additionally, achieving high bandwidth and gain are included in the objectives to minimize frequency-detuning challenges and facilitate far-field wireless communication. Accordingly, this work proposes the numerical design and fabrication of an implantable antenna resonating at a frequency of 2.442 GHz (ISM band) for brain-machine interface (BMI) technology. The key component of the antenna is a metamaterial U-shaped unit cell, serving as a radiator and connected via an inset-fed transmission line. The unit cell structure is employed to lengthen the electric path within the antenna patch, reducing its size without relying solely on the electrical properties of the MTM. Moreover, a shorting pin is used between one of the arms of the U-shaped radiator and the ground to further miniaturize the antenna size. The design utilizes a commonly available FR-4 substrate with the encapsulation of polyamide film for biocompatibility. The proposed antenna is designed using CST Studio Suite and tested through in-vivo measurements in a sheep's brain. The resulting antenna is compact in size and exhibits improved bandwidth, gain, and SAR performance. The details are organized into distinct sections: Section 2 describes the proposed design, encompassing the geometry and performance of the MTM unit cell as well as the antenna's geometry. Subsequent discussions on the antenna performances are presented in Section 3 and finally concluded in Section 4.

## 2 Proposed design

### 2.1 Metamaterial unit cell design

**Fig. 1:**
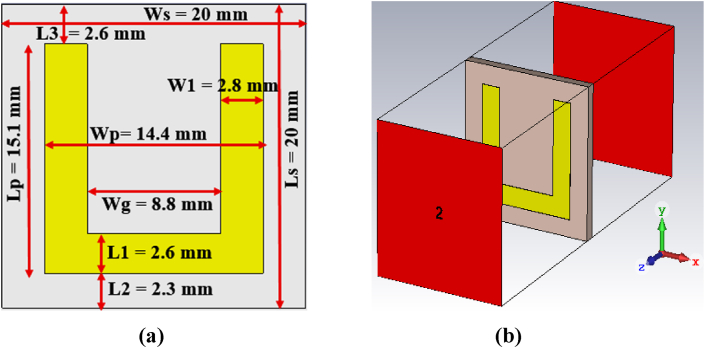
(a) Proposed MTM unit-cell, and (b) its behavior testing setup using CST tool.

Unit cell structures are fundamental building blocks used in the design of various electromagnetic structures, including antennas. These unit cells are typically designed to operate efficiently at specific frequencies determined by their geometric and material properties. Through careful design and scaling techniques, the performance of unit cell structures can also be extended to operate across different frequency bands with improved gain and bandwidth [ [19]]. However, due to the strict requirements of operating frequency and compact size, this study focuses on using an MTM unit cell connected to an inset-fed transmission line exclusively. The proposed MTM is designed in CST through numerous numerical simulations, tailoring the arms of a U-shaped structure. This process is followed by adjustments to the reflection coefficient and transmission coefficient until the desired MTM characteristics are achieved through the Nicolson-Ross-Weir (NRW) approach [ [16]]. The tailoring process is carried out on a trial-and-error basis to attain the desired performance. The top view of the proposed metamaterial unit cell is shown in Fig. 1(a). The unit cell is designed on a square-shaped FR-4 substrate of 1.5 mm thickness. The copper on the backside of the unit cell is etched to investigate its metamaterial properties. Generally, the Nicolson-Ross-Weir method is used to extract the permittivity ($\varepsilon_{r}$), permeability ($\mu_{r}$) and refractive index ($n_{r})$ of the metamaterial structure. Therefore, equations ((1), (2), (3)) are utilized for analysing the metamaterial properties of the unit cell [ [15,16]].

(1) $$\varepsilon_{r}=\left(\frac{2}{\text{jkh}}\right)\frac{1-S_{21}-{\;S}_{11}}{1+S_{21}+{\;S}_{11}}$$

(2) $$\mu_{r}=\left(\frac{2}{\text{jkh}}\right)\frac{1-S_{21}+{\;S}_{11}}{1+S_{21}-{\;S}_{11}}$$

(3) $$n_{r}=\sqrt{\varepsilon_{r}\times \mu_{r}\;}$$

where transmission coefficient and reflection coefficient are denoted as $S_{21}\;\text{and}\;S_{11}$, the wave number is represented as k = 2 π/λ and substrate thickness is denoted as h.

To check the metamaterial behaviour of the proposed unit cell, we have used CST software corresponding with Ref. [ [20]]. The proposed unit cell is placed between two wave ports on the positive and negative z-axes, as shown in Fig. 1(b). The perfect electric boundaries and magnetic boundaries are chosen as the x-axis and y-axis, respectively.

### 2.2 Performance of metamaterial unit cell

**Fig. 2:**
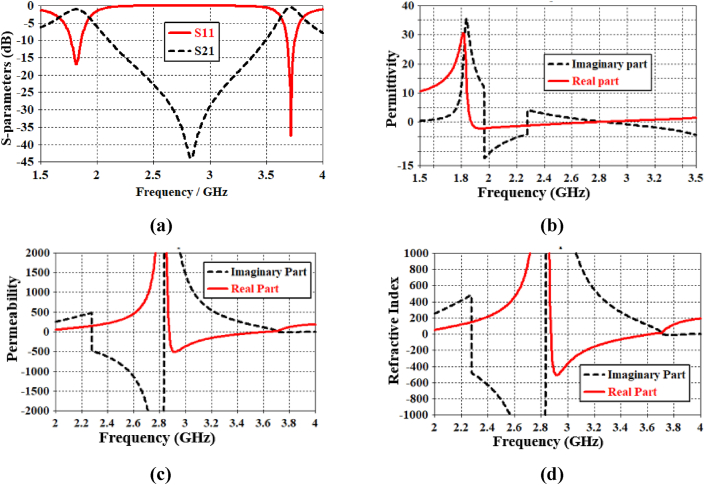
MTM unit-cell performance of (a) S-parameters, (b) permittivity, (c) permeability, and (d) refractive index.

Firstly, the reflection coefficient (S11) and transmission coefficient (S21) are extracted using the CST tool, shown in Fig. 2(a). Secondly, these values are further used to calculate the permittivity and permeability of the proposed unit cell. The real part of permittivity is shown in Fig. 2(b), which is a negative value within the frequency range of 1.87–2.65 GHz. The real part of permeability is shown in Fig. 2(c), which is also found to have a negative value within the frequency range of 2.87–3.62 GHz. Besides, the real part of the refractive index has a negative value within the range of 2.87–3.62 GHz, as shown in Fig. 2(d). According to the classification of electromagnetic material as reported in [ [21]], the proposed unit cell can be characterized as epsilon-negative (ENG) metamaterial for the ISM band (2.4–2.487 GHz).

### 2.3 Antenna design

**Fig. 3:**
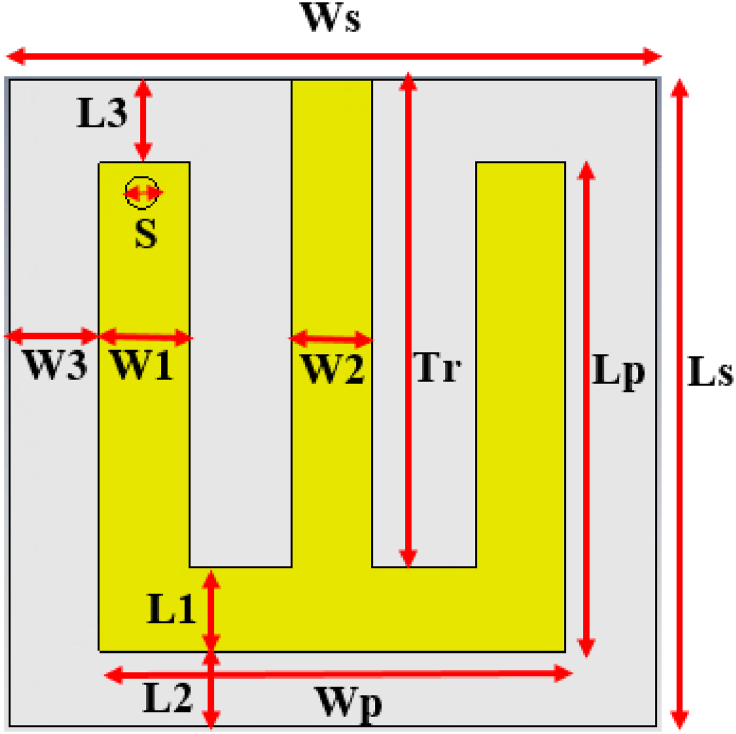
Geometry of the proposed patch antenna.

Fig. 3 shows the geometry of the antenna, which is designed on an FR-4 substrate with a relative permittivity ($\varepsilon_{r}$) of 4.3 and a loss tangent (tanδ) of 0.025. This substrate is cost-effective and readily available, making it a suitable choice for implantable antenna applications [[22], [23], [24]]. The total substrate thickness is 1.5 mm, including copper cladding on both sides, each with a thickness of 0.035 mm. Recently, flexible implantable antennas have been demonstrated as promising candidates for such applications. However, available flexible substrates have a low dielectric constant, and many of them have complex fabrication issues, particularly for compact dimensions, relatively high SAR, and demonstrated performance degradation due to bending [ [22]].

The proposed antenna is very simple with an E-shaped structure, i.e., a U-shaped unit cell is used as a radiator by connecting an inset-fed transmission line. The optimized dimensions of the antenna are 20 × 20 × 1.5 ${mm}^{3}\text{.}$ Given the fixed source impedance, substrate thickness, and dielectric constant, the width of the transmission line is manually adjusted using CST until the input impedance reaches 50 Ω. Thus, the calculated width for the inset-fed transmission line is 2.5 mm. Also, equation (4) can be utilized to verify the width of the transmission line [ [18]].

(4) $$Z=\frac{Z_{0}}{\left(\frac{W2}{t}+2\right)*\;\sqrt{\varepsilon_{r}}}$$

Here $Z_{0}$ represents the characteristic impedance (377 Ω) of the transmission line , while the input impedance of the transmission line is represented as Z. The width of the transmission line is denoted as $W_{2}$, while the permittivity and thickness of the substrate are denoted as $\varepsilon_{r}$ and t.

In addition, a shorting pin is used between one of the arms of the U-shaped radiator and ground to miniaturize the antenna size. The pin's dimension and position are manually designed, corresponding with Ref. [ [25]]. The optimized radius of the pin is 0.5 mm, and its position is (X, Y) = (−5.9, 6.5) from the center (0,0) of the antenna substrate. The ground plane of the antenna is not manipulated, and its size is the same as the substrate. Since the FR-4 substrate is not biocompatible, a very thin (0.05 mm) layer of polyamide superstrate with a relative permittivity ($\varepsilon_{r}$) of 4.3 and a loss tangent (tanδ) of 0.004 is used as biocompatible material to encapsulate the E-shaped patch and ground of the antenna, which helps to keep away the brain tissue from short circuiting. Due to its availability in different thicknesses and consistency at high temperatures, polyamide superstrate has been widely considered in numerous studies [3,14,26,27]. Therefore, the total antenna dimension is 20 × 20 × 1.6 ${mm}^{3}$ (0.338 × 0.338 × 0.027 $\lambda_{g}^{3}$). Here, $\lambda_{g}$ is the guided wavelength. The specific geometric parameters of the proposed antenna are provided in Table 1.

**Table 1:** Proposed antenna parameters.

Parameters	Value (mm)	Parameters	Value (mm)
Ws	20	W3	2.8
Ls	20	Wg	8.8
Wp	14.4	L1	2.6
Lp	15.1	L2	2.3
Tr	12.5	L3	2.6
W1	2.8	S	0.5
W2	2.5	–	–

### 2.4 Phantom design

**Fig. 4:**
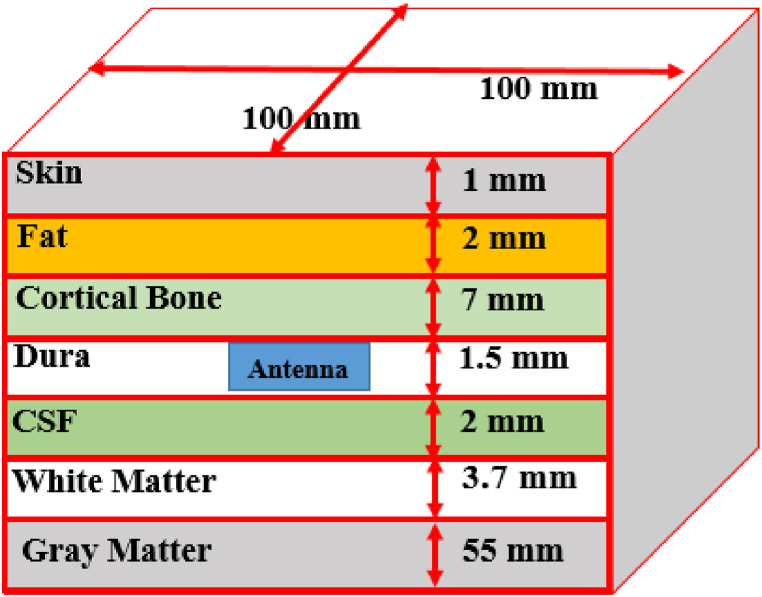
Seven-layers model of brain phantom (100 × 100 × 72.2 ${mm}^{3}$).

To assess the antenna's performance in BMI applications, it is essential to position the designed antenna within a seven-layer brain phantom. While traditional brain phantoms comprised six layers (skin, fat, cortical bone, dura, cerebrospinal fluid, and brain) for brain implantable antenna design [[28], [29], [30]], recent studies have advocated for the inclusion of seven layers. This updated model incorporates white and gray matters, each with distinct electrical properties, resulting in a more realistic brain phantom [ [8,9]]. In this study, the antenna is specifically placed between the bone and dura layers. To achieve this, a mathematical model of the seven-layer brain phantom is developed in CST. A sizable phantom dimension of 100 × 100 × 72 ${mm}^{3}$ is chosen for the investigations, as illustrated in Fig. 4. Besides, Table 2 presents the relative permittivity ($\varepsilon_{r}$) and loss tangent (tanδ) values for the seven-layer human brain tissues [ [5]]. These electrical properties are employed to create the mathematical model in CST.

**Table 2:** Tissue properties at 2.45 GHz.

Tissues	Relative permittivity	Loss tangent
Skin	42.923	0.273
Fat	5.285	0.145
Cortical Bone	11.410	0.252
Dura	42.099	0.292
CSF	66.319	0.385
Gray Matter	48.994	0.271
White Matter	36.226	0.246

## 3 Discussions of antenna performances

### 3.1 Analysis of simulation results

**Fig. 5:**
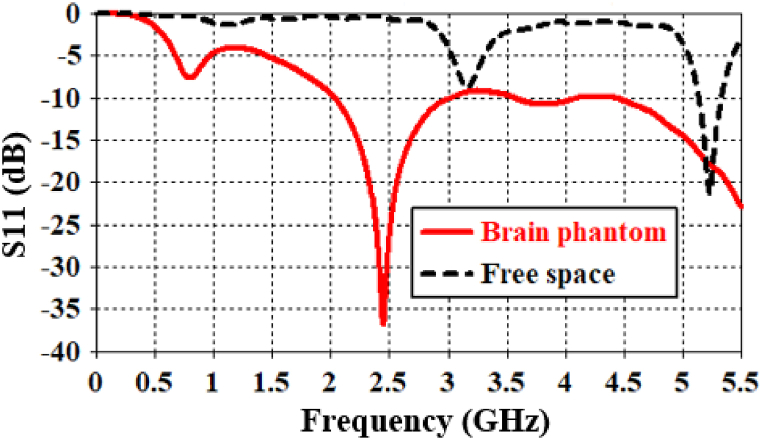
Simulated return loss parameters in both free space and inside a brain-phantom.

**Fig. 6:**
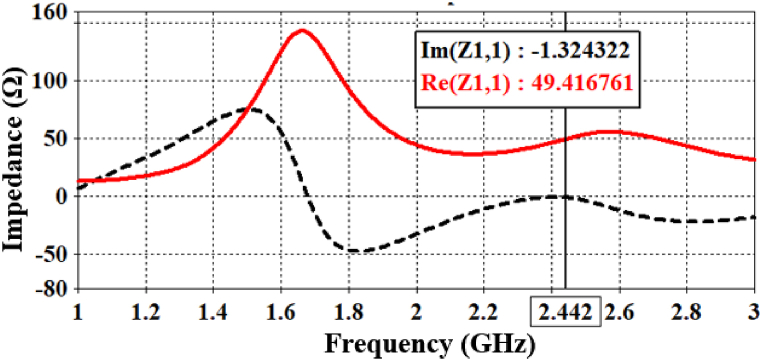
Antenna input-impedance (real and imaginary parts) at the 2.442 GHz band.

The proposed antenna operates at 2.442 GHz within the brain phantom, as illustrated in Fig. 5. It demonstrates a bandwidth of 974 MHz and a return loss of −36.74 dB. In free space, the antenna is simulated in the frequency range of 0–6 GHz, as the center frequency is shifted to 5.23 GHz. A very good return loss of −21.19 dB is observed in free-space conditions. While two additional peak resonance frequencies are present in free space, they are both below −10 dB and deemed insignificant. Furthermore, the designed antenna exhibits a very good impedance of 49.41 - j1.32 Ω inside the brain phantom, as depicted in Fig. 6.

**Fig. 7:**
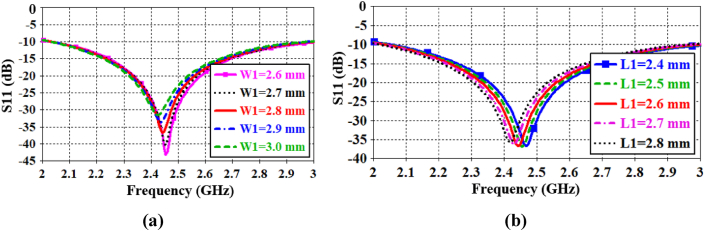
Parametric analysis of the reflection coefficient for (a) different widths of W1, and (b) different lengths of L1.

In this work, some parametric performances are evaluated to demonstrate the fabrication tolerance of the antenna. Since the transmission line is connected with the U-shaped MTM, the width and length (W1 and L1) of the U-shaped MTM are assessed. When the value of W1 is varied from 2.6 to 3 mm, the resonance frequencies remain within the ISM band, as shown in Fig. 7(a). Similarly, when the value of L1 is adjusted from 2.4 to 2.8 mm, the resonance frequencies still fall within the ISM band, as depicted in Fig. 7(b). Therefore, it is evident that fractional changes in the width and length of the U-shaped radiator do not significantly affect the antenna's performance. The optimized values of W1 and L1 are determined to be 2.8 mm and 2.6 mm, respectively.

**Fig. 8:**
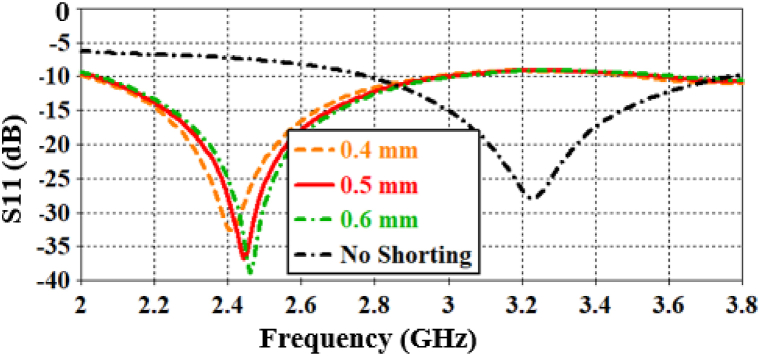
Influence of the diameter of the shorting pin on the return loss.

The shorting pin assists in designing electrically small antennas that operate at the same resonance frequency as those supposed to resonate in larger antennas [ [9]]. The proposed antenna is entirely dependent on the shorting pin to resonate in the ISM band at 2.442 GHz. According to Fig. 8, different pin radii are investigated to demonstrate the effect of the shorting pin. With an increase in radius, the resonance frequency shifts to the right; otherwise, it shifts to the left. Minor changes in the pin radius do not significantly affect the antenna's resonance frequency. The optimized radius of the shorting pin is found to be 0.5 mm. On the other hand, the antenna operates in the UWB band at 3.2 GHz without the shorting pin, and this frequency band is permissible for tissue implantation [ [14]]. In this case, the bandwidth is found to be 991 MHz. In the context of antenna design, introducing a shorting pin can indeed extend the effective electrical length of the structure, causing a phase delay in the signal. This delay effectively slows down the signal, leading to a change in the resonance frequency and wavelength of the antenna. Therefore, with the shorting pin, the antenna resonates in the 2.442 GHz band instead of the 3.2 GHz band.

**Fig. 9:**
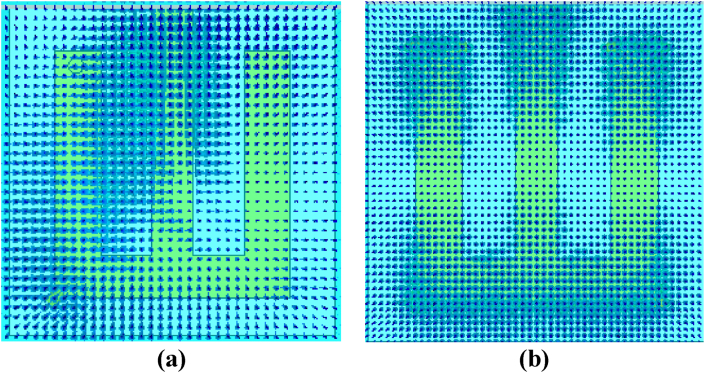
Electric field distribution on the patch for the cases of (a) with a shorting pin, and (b) without a shorting pin.

The phase delay is a result of the altered path that the signal takes due to the shorting pin, and it experiences a change in impedance and a corresponding phase shift. This phase shift directly affects the electric-field distribution along the structure. According to Fig. 9(a), the antenna shows a much higher electric-field distribution near the shorting pin position, i.e., around the left side of the antenna patch compared to the right side. Conversely, the electric-field distribution is the same on both sides of the antenna patch when the antenna performs in the UWB band of 3.2 GHz without a shorting pin, as illustrated in Fig. 9(b).

**Fig. 10:**
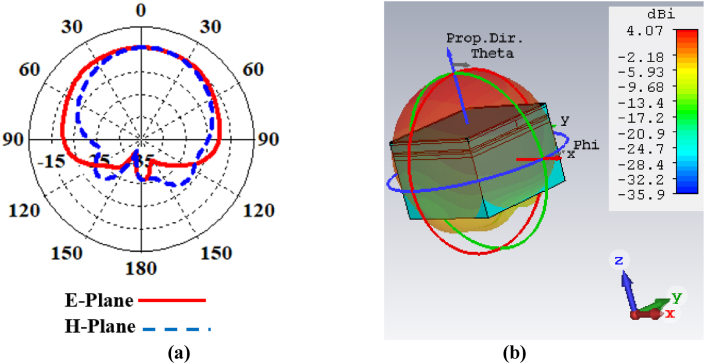
Investigation of (a) polar radiation pattern, and (b) directivity pattern of the proposed antenna.

Fig. 10(a) depicts the polar radiation patterns of the proposed antenna. Even with side lobes of −11.4 dB and −6.7 dB, the antenna displays a directed radiation pattern in the E-plane and H-plane at 2.442 GHz. To minimize interference effects during communication with the receiver antenna, a directive radiation pattern is better than an omni-directional antenna [ [5,7]]. As the side lobe is not essential for BMI applications, these small side lobes will have less effect inside the brain tissue. According to Fig. 10(b), the antenna directivity is found to be 4.07 dBi, and it shows the direction of the main lobe towards the +z direction.

**Fig. 11:**
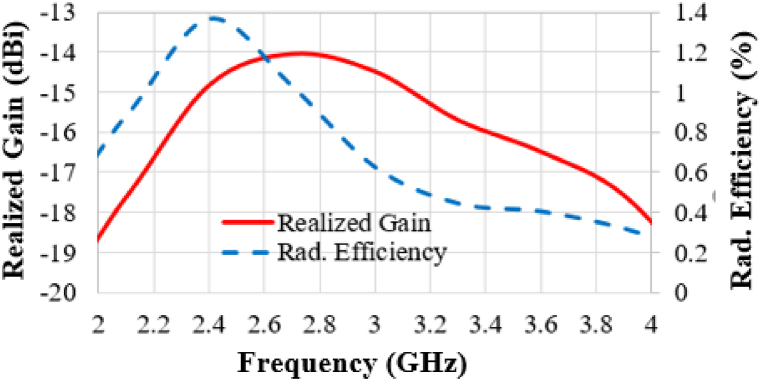
Radiation efficiency and the realized gain as a function of frequency.

When an antenna is implanted within the complex structure of brain tissue, it interacts with the surrounding electromagnetic fields. This phenomenon occurs because brain tissue, composed of various layers, exhibits different electromagnetic properties that can absorb electromagnetic waves. As a result, antennas implanted in such environments typically exhibit low radiation efficiencies. As shown in Fig. 11, the radiation efficiency is found to be 1.32% in the 2.442 GHz band. These values are particularly relevant when considering applications involving low-power transmission. Notably, this observed efficiency is better than that of many other implantable antennas operating in the 2.4 GHz band, as documented in previous research studies, e.g., [ [5,9]]. Besides, the gain of the antenna is found to be −14.6 dBi in the 2.442 GHz band. Implantable antennas often provide low gain, as illustrated in Table 5, due to their small aperture and the surrounding lossy implant.

### 3.2 Effects of the MTM unit-cell

**Fig. 12:**
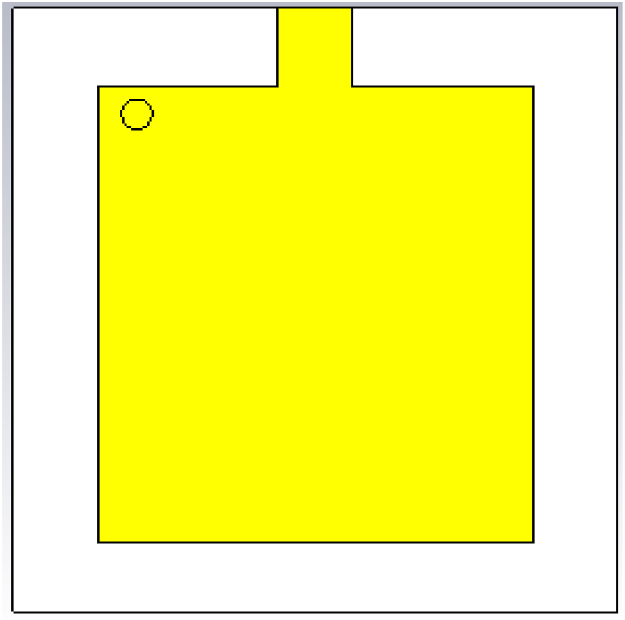
Antenna patch without the U-shaped MTM radiator.

**Fig. 13:**
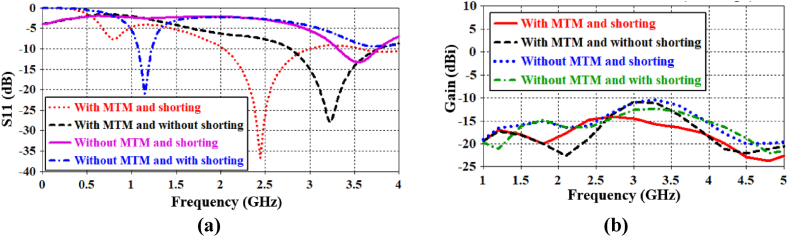
Comparisons of (a) reflection coefficients, and (b) antenna gains among different antenna structures.

To investigate the effect of the U-shaped MTM unit cell as an antenna radiator, we assumed the antenna structure was rectangular-shaped with a shorting pin, according to Fig. 12. It is designed (Fig. 12) utilizing the proposed U-shaped radiator (Fig. 3) covered with copper cladding so that no gap remains inside the radiator. Fig. 13(a) illustrates the antenna performances (resonance frequency, bandwidth, and gain) among different structures. Particularly, the influences of the MTM unit cell and the shorting pin are explicitly investigated here. This figure shows that the antenna resonates at 2.442 GHz with a bandwidth of 974 MHz and a gain of −14.6 dBi considering both the MTM unit cell and the shorting. The antenna with the same MTM unit cell but without shorting resonates at 3.2 GHz with a bandwidth of 991 MHz and a gain of −11 dBi. So, the shorting pin has a small effect on the gain, and its effect is insignificant on the bandwidth. However, its operating frequency as well as bandwidth are significantly affected by the presence of MTM unit cell. The antenna resonates at 1.146 GHz with a bandwidth of 154 MHz and a gain of −20.6 dBi when there is no MTM unit cell and with shorting (Fig. 12). Again, it resonates at 3.55 GHz with a bandwidth of 454 MHz and a gain of −11.4 dBi in the absence of MTM unit-cell and shorting. Therefore, these discussions demonstrate that the proposed design achieves a high bandwidth by employing an MTM unit cell as a radiator. Besides, the effect of the MTM unit cell on antenna gain is illustrated in Fig. 13(b). It is described that the MTM unit cell has a limited impact on antenna gain, but the overall gain performance at 2.442 GHz and 3.2 GHz bands is quite good, particularly considering the small size of the antenna.

### 3.3 Effect of implantable device on antenna performances

**Fig. 14:**
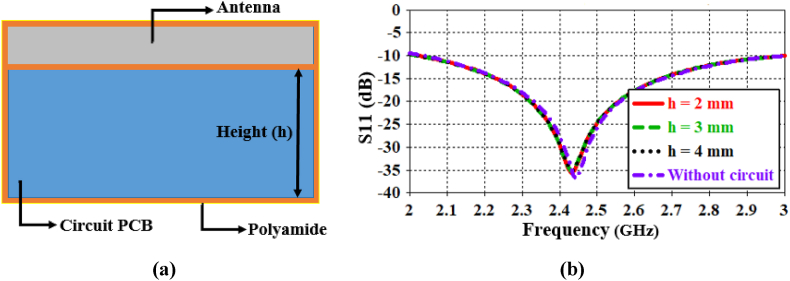
(a) The proposed antenna with a PCB circuit coated with polyamide, and (b) the effect of the PCB circuit on antenna performance.

The brain implantable device mainly consists of an antenna, data package, sensors, power management, batteries, and IMD casing. To validate the proposed antenna performance, a PCB circuit is required to be connected underneath the antenna. Therefore, a perfect electric conductor (PEC) material is considered in place of the PCB circuit, which is in agreement with Ref. [ [2,12]]. As depicted in Fig. 14(a), the whole system is encapsulated with a biocompatible polyamide casing. The thickness of the polyamide layer is 0.05 mm. The length and width of the casing are kept the same as the antenna. In this system, the antenna's patch is covered by the upper layer of the polyamide casing. Additionally, the polyamide layer is used to coat the ground of the antenna, where the PEC material is attached underneath the layer. To assess the effect of the PEC material, different heights of it are examined, as shown in Fig. 14(b). The heights are varied up to 4 mm, and they do not significantly affect the antenna's performance. The resonance frequency remains within the ISM band of 2.43 GHz in each case, whereas without the circuit, it resonates at the 2.442 GHz band.

### 3.4 Specific absorption rate (SAR) calculation

**Fig. 15:**
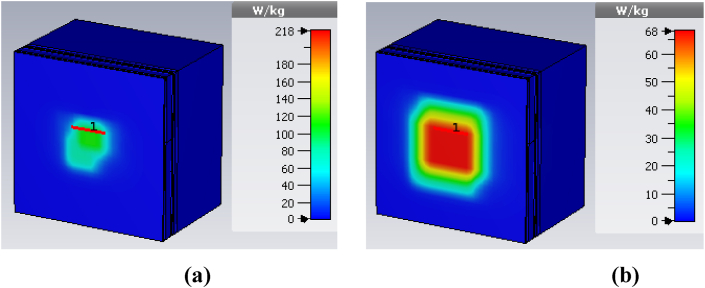
SAR calculation for (a) 1g-standard, and (b) 10g-standard.

There are two standards for specific absorption rate (SAR) calculation: the 1g average standard and the 10g average standard. SAR values must adhere to FCC and ERC standards of IEEE compliance, with limits set at 1.6 W/kg for 1 g of tissue mass and 2 W/kg for 10 g of tissue mass [ [8,9]]. Additionally, the effective isotropic radiated power (EIRP) for wireless telemetry at 2400 MHz is limited to 100 mW (20 dBm) by FCC and ETSI standards [ [9]]. We used 1W of input power to calculate the SAR of the proposed antenna inside a seven-layer brain phantom. The calculated SAR is found to be 218 W/kg and 68 W/kg for 1g and 10g tissue standards respectively, as shown in Fig. 15 (a and b). According to Table 3, it is clear that the safety input power should be between 7.3 mW and 29.4 mW to stay within the SAR limits.

**Table 3:** SAR calculation at 2.442 GHz.

Tissue standard	Max. average ${SAR}_{W/\mathrm{kg}}$	Max. allowable input power (mW)
1g-average	218	7.3
10g-average	68	29.4

### 3.5 Link budget calculation

To establish far-field communication between the transmitter ($T_{x}$) and receiver ($R_{x}$), it is essential to calculate the link budget for perfect communication. The following equations (5), (6) can be used to calculate the link budget [ [12,31]].

(5) $$\text{Link}\;\text{Margin}\;\left(\text{dB}\right)=P_{t}+G_{t}‐L_{f}+G_{r}‐N_{0}‐E_{b}/N_{0}‐10\;\log_{10}B_{r}+G_{c}‐\;G_{d}$$

(6) $$\text{Free}\;\text{space}\;\text{path}\;\text{loss},L_{f}=20\;\log_{10}\frac{4\pi d}{\lambda }$$

Where transmitted power and transmitter antenna gain are denoted as $P_{t}$ and $G_{t}$, respectively. The receiver antenna gain and noise power density are denoted as $G_{r}$ and $N_{0}$. For signal quality measurement, bit rate is denoted as $B_{r}$, and free space path loss is denoted as $L_{f}$. The distance between the transmitter and receiver antenna is denoted as d. To simplify the design, it is not taken into consideration the losses related to polarization and impedance mismatch. A linearly polarized dipole antenna is assumed to be a receiver antenna whose gain is 2.15 dBi. The details of the link budget parameters are listed in Table 4.

**Table 4:** Link budget parameters.

Transmitter specifications (proposed antenna)	
Central frequency	2442 MHz
Transmitted power ($P_{t}$)	−40 dBW (100 μW)
Gain ($G_{t}$)	−14.6 dBi
EIRP	−54.6 dBW
**Receiver specifications (assumed)**	
Gain $(G_{r}$)	2.15 dBi
Temperature (T)	293 K
Boltzmann constant (K)	−1.38 × $10^{-23}$
Noise power density (No)	−203.93 dB/Hz
**Signal quality (assumed)**	
Bit rate ($B_{r}$)	1 Mbps
Bit error rate	1 × $10^{-5}$
Ideal PSK (Eb/No)	9.6 dB
Coding gain ($G_{c}$)	0 dB
Fixing deterioration ($G_{d}$))	2.5 dB

**Table 5:** Performance comparison with recent literature.

Ref./Year	Freq. (MHz) /Patch structure	Implant tissue	Volume ($\lambda_{g}^{3}$)	BW (%)	Gain (dBi)	SAR (W/kg) @ 1 W i/p power	
						1-g	10-g
[ [36]] /2016	402/2400	Skin	0.562 × 0.588 × 0.0325	7.4/6.6	−36.7/-27.1	832/690	–
[ [37]] /2019	2480	Muscle	0.371 × 0.340 × 0.0011	24.19	−19.7	0.914	–
[ [38]] /2020	2490	Muscle	0.505 × 0.488 × 0.028	7.35	4.813	–	–
[ [39]]/2021	2450	Brain, Skin	0.266 × 0.225 × 0.05	13.7	−17.08	900	82.1
[ [40]]/2022	2450	Skin	1.305 × 1.553 × 0.016	–	−14.7	1.8	0.3
[ [41]]/2023	2450	Skin	0.261 × 0.261 × 0.0668	4.8	−18.41	545	–
**This Work**	**2442**	**Brain**	**0.338 × 0.338 × 0.027**	**39.89**	−**14.6**	**218**	**68**

**Fig. 16:**
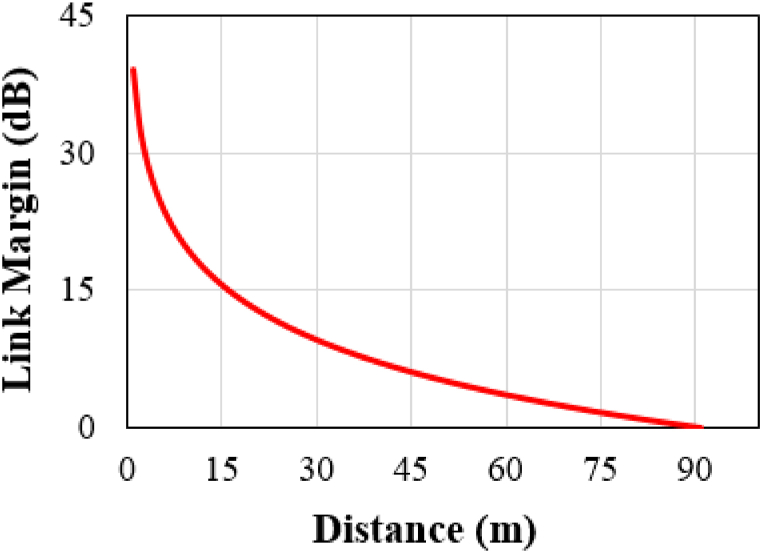
Link budget at 1 Mbps data speed in between transmitter ($T_{x}$) and receiver ($R_{x}$) antennas.

It is sufficient for an implantable antenna to transmit data at speeds of $B_{r}$ = 7 Kbps or 100 Kbps [ [32]]. Additionally, a link margin of 15 dB is considered adequate for practical applications [ [12]]. In this study, high data rates of 1 Mbps are used to calculate the link margin, as shown in Fig. 16. Since high data rates and low power (complying with the ERC limit) result in low-range wireless communication, the proposed antenna can transmit data to the receiver from an approximate 15-m distance. In this scenario, the FCC and ETSI suggested an EIRP limit of 20 dBm at 2.45 GHz, which exceeds our proposed calculated EIRP of −24.6 dBm (−54.6 dBW).

### 3.6 Antenna fabrication and testing

**Fig. 17:**
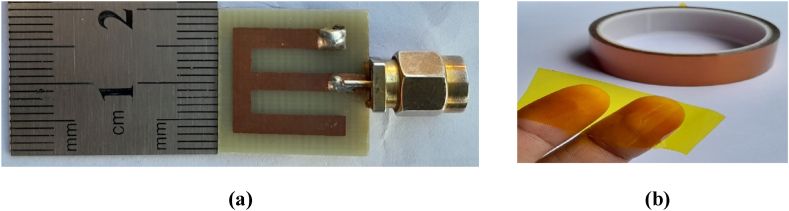
Pictorial view of the (a) fabricated antenna next to a ruler, and (b) biocompatible material (polyamide).

The proposed antenna is fabricated as shown in Fig. 17(a) using the lithography technique. For the shorting pin, it is drilled with a 0.5 mm radius in the exact designed position. The shorting is made with copper wire, and it is soldered with lead wire due to its availability and good electrical conductivity. Additionally, the antenna is soldered with an SMA male connector to feed the inset-fed transmission line. This soldering material introduced an air gap during the encapsulation of the antenna with a biocompatible superstrate. A biocompatible polyamide superstrate, illustrated in Fig. 17(b)–is applied to both sides of the patch and ground. This superstrate is 0.05 mm thick and appears transparent due to its very small thickness.

**Fig. 18:**
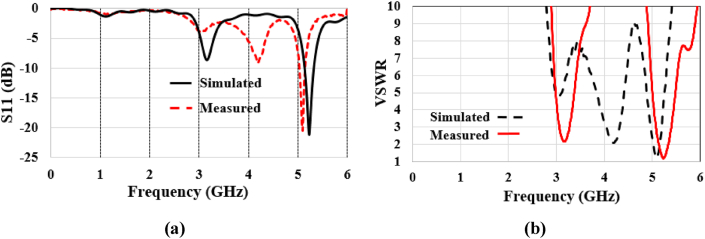
Simulated and measured results in free space: (a) reflection coefficients, and (b) VSWR.

Anritsu VNA Master MS2036C is used for measurement of the proposed antenna [ [33]]. The reflection coefficient is measured in free space conditions, and then it is compared with the simulation performance, as shown in Fig. 18(a). The reflection coefficient is found to be −20.6 dB at 5.23 GHz in the simulation test and 22.5 dB at 5.13 GHz in the measurement test. These results demonstrate almost identical antenna performance between simulation and measurement. To investigate the mismatch between the source and load circuits, the voltage-standing wave ratio (VSWR) is measured. As depicted in Fig. 18(b), the experimental results closely match the simulation, where VSWR is simulated as 1.19 at 5.23 GHz and measured as 1.22 at the 5.13 GHz band. It indicates that the mismatch loss is near 0 dB.

**Fig. 19:**
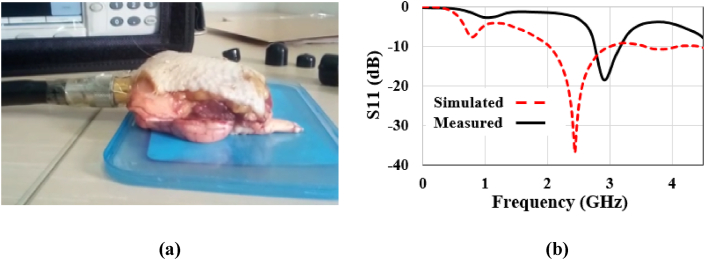
Investigation of (a) in-vivo measurement setup, and (b) reflection-coefficient parameters inside seven-layer brain tissues.

For in-vivo testing of such implantable antennas, since this area of ongoing research has no specific standard measurement method, researchers commonly use artificial tissue phantoms for in-vitro measurements [5,8,9] and animal tissues for in-vivo measurements [ [12,34]]. However, the electrical properties of artificial tissue phantoms may change with temperature and over time [ [35]]. The challenges may also arise from differences in the electrical properties of human and animal brain tissues. In this study, the proposed antenna is tested using animal tissues, following the approach outlined in [ [34]]. Skin tissue is obtained from a deceased chicken, fat tissue from a deceased goat, and cortical bone and brain tissue from a deceased sheep. The in-vivo measurement procedure is depicted in Fig. 19(a), where the Vector Network Analyzer (VNA) and antenna are connected through a coaxial waveguide port for measurement purposes.

Fig. 19(b) shows the simulated and experimental results for the prototype antenna. It shows a little shift in resonance frequency, which is 2.507 GHz in the measurement test in comparison to the simulated 2.442 GHz. This is possibly due to an air gap between the antenna and superstrate, and soldering issues with the shorting pin and SMA connector. In addition, the proposed phantom is rectangular in shape (100 × 100 × 72.2 ${mm}^{3}$) which is similar to that used in Refs. [[5], [6], [7], [8]], while a sheep's brain is smaller, spherical, and not flat. The difference in the size of the analysis domain may also lead to a minimal shift in resonance frequency when comparing simulation and experimental results. Additionally, the insertion of a waveguide port between the cortical bone and the brain surface during measurement introduces an air gap, contributing to the observed differences between simulation and experimental results. Despite these factors, the shape of the S-parameter curve closely resembles the simulation curve. As the design incorporates a U-shaped structure with an inset-fed transmission line, there is potential for further meandering of the structure to minimize the antenna size. Moreover, the ground is not manipulated; therefore, a defected ground structure will be considered to further reduce its size.

### 3.7 Performance analysis of the proposed antenna

The performance of the designed antenna is compared with some recent relevant literature in Table 5. The proposed antenna is found to be relatively compact compared to many literatures [[36], [37], [38],^40^] and offers a high bandwidth, which are important requirements for implantable medical devices. It also exhibits comparatively high gain and low SAR. The proposed antenna patch has a simpler structural design, and the ground plane is not modified. Such a simple design shows high fabrication tolerance, since fabrication tolerance usually reduces with design complexity. Furthermore, as the proposed antenna is designed to operate under cortical bone tissue, there is a potential opportunity to expand its functionality to deeper tissues, including skin, fat, and muscle. Therefore, further development can be carried out to assess the antenna's performance in such deep tissue layers.

## 4 Conclusion

A compact and high performance microstrip patch antenna has been proposed in this study for Brain-Machine Interface (BMI) technology. The key component of the antenna was a U-shaped metamaterial (MTM) unit cell, which was connected by an inset-fed transmission line. In contrast to relying on the electrical characteristics of the MTM, the concept of this study was to utilize the MTM unit cell as a patch radiator, introducing a unit-cell structure to extend the electric path within the antenna patch. This technique helped effectively reduce the antenna size as well as improve the bandwidth. Additionally, a strategically placed shorting pin between one of the arms of the U-shaped radiator and the ground proved instrumental in achieving a compact antenna size of 20 × 20 × 1.6 ${mm}^{3}$ (0.338 × 0.338 × 0.027 $\lambda_{g}^{3}$) and resonance at the 2.442 GHz band within a seven-layer brain phantom. The proposed antenna exhibited a high bandwidth of 974 MHz and a gain of −14.6 dBi at the 2.442 GHz band. Moreover, the specific absorption rate (SAR) was investigated, revealing values of 218 W/kg and 68 W/kg for 1g and 2g tissue standards, respectively. Therefore, to adhere to FCC standards, the corresponding maximum allowable power should be between 7.3 and 29.4 mW. Furthermore, an input power of −40 dBW (100 μW) was chosen for the antenna to calculate the link budget, ensuring that the specific absorption rate (SAR) remains within acceptable limits. Consequently, the proposed transmitter antenna can establish communication with the receiver antenna at a distance of approximately 15 m, maintaining an acceptable 15 dB link margin. The antenna was fabricated and measured in both free space and in-vivo conditions using sheep's brain. In the measurements, the resonance frequency in free space and corresponding VSWR were closely aligned with the simulation results. However, in the case of in-vivo measurements, while the S-parameter curve was nearly identical, a minor discrepancy was noted between the simulation (2.442 GHz) and experimental (2.507 GHz) resonance frequencies. This divergence can be attributed to challenges related to air gaps and the use of animal brain tissues. In addition, the antenna's performances were benchmarked against recently published works, demonstrating superior bandwidth, gain, and specific absorption rate (SAR) characteristics.

## Data availability

No data were used for the research presented in this paper.

## CRediT authorship contribution statement

**Emtiaz Ahmed Mainul:** Writing – review & editing, Writing – original draft, Methodology, Investigation, Formal analysis, Data curation, Conceptualization. **Md Faruque Hossain:** Writing – review & editing, Supervision, Methodology, Investigation, Formal analysis, Conceptualization.

## Declaration of competing interest

The authors declare that they have no known competing financial interests or personal relationships that could have appeared to influence the work reported in this paper.
